# Variations in the Origin and Communication of the Intercostobrachial Nerve: Surgical Implications

**DOI:** 10.7759/cureus.113762

**Published:** 2026-08-01

**Authors:** Sarika R Tigga, Sandeep Saluja

**Affiliations:** 1 Anatomy, University College of Medical Sciences, Guru Teg Bahadur Hospital, New Delhi, IND; 2 Anatomy, Amrita School of Medicine, Faridabad, Faridabad, IND

**Keywords:** axilla, intercostal nerve, intercostal space, intercostobrachial nerve, mastectomy, medial cutaneous nerve of arm

## Abstract

The intercostobrachial nerve (ICBN) supplies the upper part of the postero-medial region of the arm, lateral thoracic wall, and axilla. It is susceptible to being damaged during various axillary surgeries like axillary lymph node dissection, sentinel lymph node biopsy, and mastectomy because of its diverse origin and branching configuration. Safeguarding the ICBN during axillary surgical procedures can minimise postoperative discomfort and paraesthesia.

During standard dissection of the right side axilla and pectoral region of a female cadaver, the ICBN was detected, and its path was followed. The normally originating ICBN was observed to split into two branches after emerging from the second intercostal space (ICS). The lateral cutaneous branch of the third intercostal nerve emerged from the third ICS as a variant ICBN and joined with the lower branch of the ICBN. Subsequently, both the ICBNs supplied the upper one-third of the posteromedial region of the right arm. The ICBNs were not observed to be linked with the medial cutaneous nerve of the arm (MCNA). Furthermore, the medial cord of the right brachial plexus provided a combined trunk, which split at the middle of the arm as the MCNA and median cutaneous nerve of the forearm.

The variant site of origin of the ICBN from the third ICS and its communication with the normally originating ICBN, as seen in the present case, may enhance the likelihood of its injury during several operative procedures such as mastectomy, breast reconstruction, and axillary lymph node dissection. Thorough awareness of such cases of diverse origin and communication of the ICBNs, combined with careful surgical approaches, can potentially help in the reduction of post-surgical pain and paraesthesia.

## Introduction

The intercostobrachial nerve (ICBN) is the lateral cutaneous branch of the second intercostal nerve (ICN). The ICBN runs obliquely from the second intercostal space (ICS) down the axilla, passing via the central axillary lymph nodes. It forms a connection with the medial cutaneous nerve of the arm (MCNA) in the axilla. Subsequently, it penetrates the deep fascia of the arm and innervates the upper half of the postero-medial aspect of the arm, where it communicates with the posterior brachial cutaneous branch of the radial nerve. Its size is inversely proportionate to that of the MCNA. Additionally, it also innervates the lateral thoracic wall and the axilla [1].

The passage of the ICBN in connection with the axilla presents a hazard of iatrogenic harm ensuing from routine operating approaches like axillary lymph node dissection, sentinel lymph node biopsy, and mastectomy. The ICBN is the most frequently affected nerve during mastectomy and is alleged to be involved in prolonged pain after breast cancer surgery as well as irreversible impairment of sensory function in the postero-medial side of the arm [2]. The ICBN safeguarding may reduce postoperative dysaesthesia, paraesthesia, and discomfort while additionally boosting quality of life. Surgeons must be very careful when operating in the axillary region, as the ICBN's early divisions and connections to the brachial plexus may be at risk [3].

The structure of the ICBN is remarkably diverse, with multiple origins, variant courses and interconnecting branches. Identifying the exact frequency and various forms of origin and branching patterns of ICBN would help surgeons to secure these neuronal structures during surgical management of breast cancer [2]. Despite anatomical variances, ICBN could be a reasonable option for neurotization to lateral and medial contributions of the median nerve in the recovery from sensory injury to the hand. The ICBN neurotization appears to be feasible, as the employment of other donor nerves is not only challenging but also has lower sensory restoration prospects. Another advantage is that the cortical topography of the ICBN is close to the hand area [4].

The clinical relevance of the ICBN demands further research in the coming years because, despite the development of comprehensive anatomical investigations, the variant ICBN with the coexistence of the other common variations remains infrequently documented [2]. Recognising the significance of understanding the normal and variant architecture of the ICBN in operating procedures of the breast and axilla, we present a case of unilateral variant origin of the ICBN from the lateral cutaneous branch of the third ICN connecting with the normally originating ICBN from the lateral cutaneous branch of the second ICN and coexistence of a common trunk for the MCNA and the medial cutaneous nerve of the forearm (MCNF).

This work was earlier submitted as a conference abstract/poster at the 71st National Conference of the Anatomical Society of India, Sri Guru Ram Das Institute of Medical Sciences and Research, Sri Amritsar, on 22-24 November 2024.

## Case presentation

**Figure 1:**
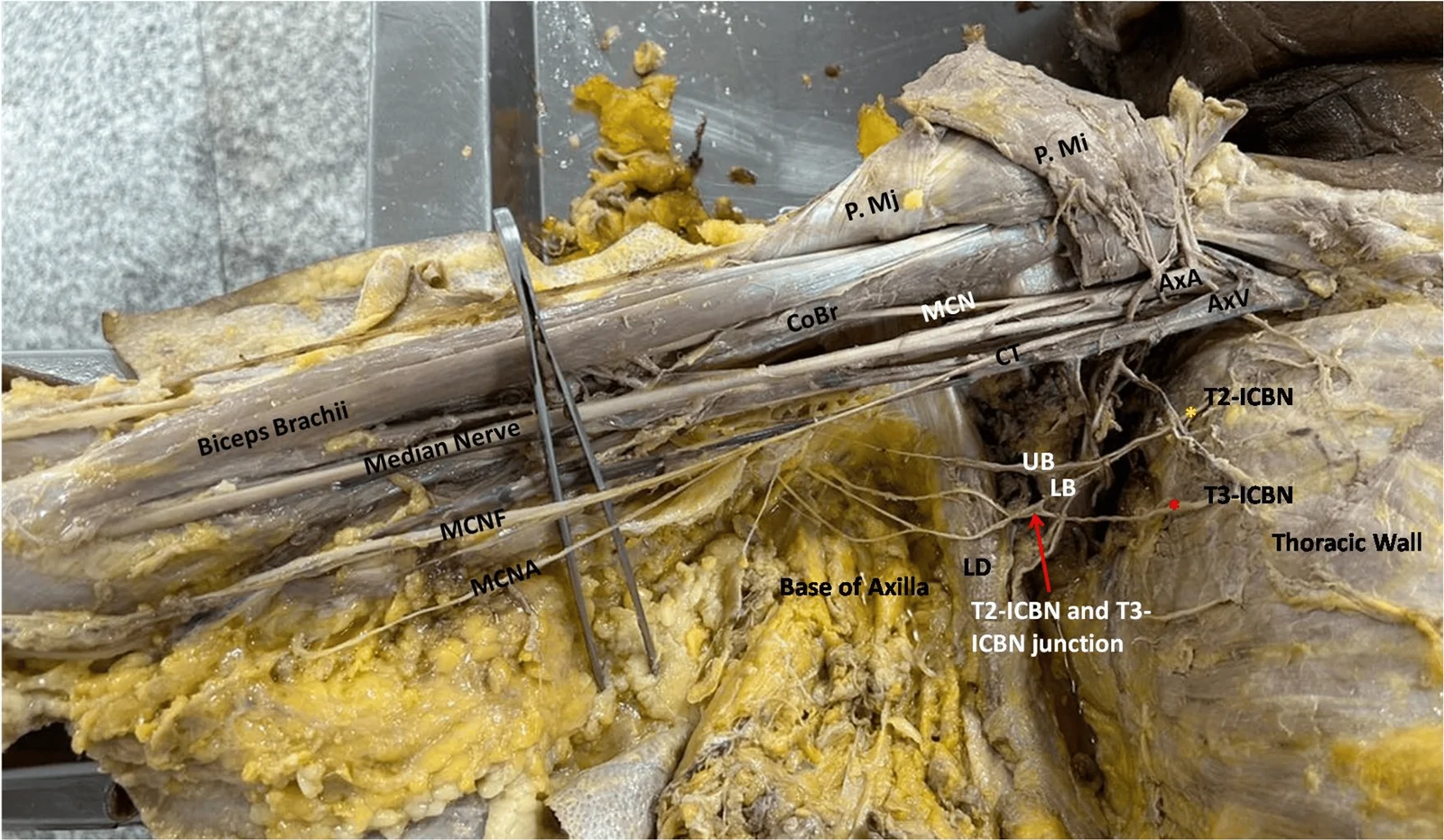
Dissected right axillary region showing the origin of the intercostobrachial nerve (ICBN) from the second intercostal space (T2-ICBN), its upper (UB) and lower (LB) branches, ICBN of the third intercostal space (T3-ICBN), the common trunk (CT) for the medial cutaneous of the arm (MCNA) and the medial cutaneous of the forearm (MCNF) P. Mi: pectoralis minor; P. Mj: pectoralis major; CoBr: coracobrachialis; LD: latissimus dorsi; AxA: axillary artery; AxV: axillary vein; MCN: musculocutaneous nerve; T2-ICBN: lateral cutaneous branch of the second intercostal nerve; T3-ICBN: lateral cutaneous branch of the third intercostal nerve; T2-ICBN and T3-ICBN junction shown by red arrow; T2-ICBN shown by yellow asterisk; T3-ICBN shown by red asterisk

During standard dissection of the right axilla of a 75-year-old female cadaver, the anomalous origin and communication of the ICBN were discovered. The normally originating ICBN (T2-ICBN) was identified as emerging from the second ICS, which split into upper and lower branches. The lower branch of the T2-ICBN was linked with the lateral cutaneous branch of the third ICN (T3-ICBN), which was identified as the variant origin of the ICBN. Both branches were distributed to the upper one-third of the postero-medial region of the right arm after splitting into smaller branches. There was no connection between the ICBN and the MCNA (Figure 1).

**Figure 2:**
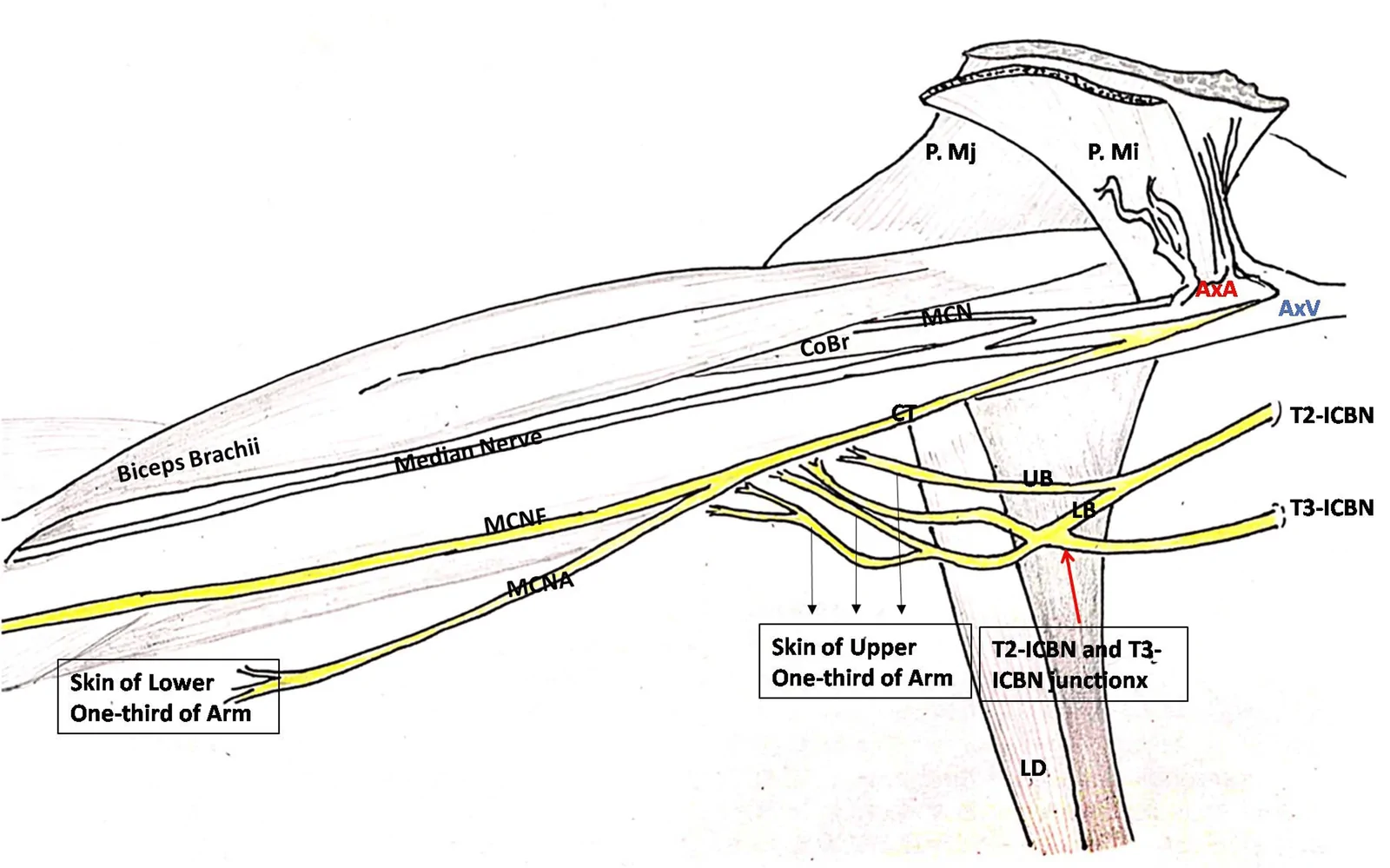
Diagram of the right axillary region It shows the origin of the intercostobrachial nerve (ICBN) from the second intercostal space (T2-ICBN), its upper (UB) and lower (LB) branches, ICBN of the third intercostal space (T3-ICBN), the common trunk (CT) for the medial cutaneous of the arm (MCNA) and the medial cutaneous of the forearm (MCNF). P. Mi: pectoralis minor; P. Mj: pectoralis major; CoBr: coracobrachialis; LD: latissimus dorsi; AxA: axillary artery; AxV: axillary vein; MCN: musculocutaneous nerve; T2-ICBN: lateral cutaneous branch of the second intercostal nerve; T3-ICBN: lateral cutaneous branch of the third intercostal nerve; T2-ICBN and T3-ICBN junction shown by red arrow Image credit: Hand-drawn diagram created by the authors and labelled after transferring into Microsoft PowerPoint (Microsoft Corp., Redmond, WA, USA).

Additionally, on further dissection of the right arm, it was observed that the medial cord of the right brachial plexus provided a common trunk which originated deep to the pectoralis minor muscle just above the medial root of the median nerve. The common trunk bifurcated at mid-arm level as the MCNA and the MCNF. The MCNA distributed to the inferior one-third of the medial side of the arm, and the MCNF followed its normal course by providing branches to the medial side of the forearm. However, the left ICBN was observed to be as usual in its origin, course, and termination, and there was no common trunk for MCNA and MCNF on the left side (Figures 1, 2).

## Discussion

The present case displays unique variability in the origin and branching configuration of the ICBN. After arising from the second ICS, the normally originating ICBN bifurcated, and its lower branch joined with the variant ICBN emerging from the third ICS without any MCNA communication. Additionally, a common trunk was observed for the MCNA and the MCNF. Unawareness of the unique coexistence of these findings may enhance susceptibility to damage to the ICBN and the common trunk during various axillary surgeries, and their disruption may be responsible for post-procedural discomfort, paraesthesia, and a decrease in overall quality of life [3].

During a standard dissection of the axilla in a 75-year-old female cadaver, Siddiqui et al. observed an inadequately documented variety of the ICBN, where it passed through the second ICS as a unified trunk and subsequently separated into two distinct branches [5]. Similarly, Rustagi et al. discovered a thick ICBN arising typically from the second ICS during dissection of the right axilla in an adult male cadaver. Furthermore, in addition to the ICBN, two nerves, ICBN1 and ICBN2, arose from the second ICS and travelled to the medial side of the arm, connecting with MCNA 2 and MCNA 1, respectively. These regional neuronal links between the ICBN and the branches of the medial cord can lead to sensory deficits subsequent to various axillary surgeries such as mastectomy or long thoracic nerve exploration. Furthermore, they believed that additional nerves originating from the second ICS could be employed for nerve graft surgeries [6]. However, the current study did not demonstrate the presence of an additional ICBN arising from the second ICS, nor did it reveal any communication with the MCNA.

Singh et al., during dissection of the right axilla, upper arm, and pectoral region, noticed that the lateral cutaneous branch of the second ICN was carrying both sensory and motor parts. The motor part innervated both the pectoralis major and minor, while the sensory part innervated the axillary floor and the medial aspect of the arm. On the left side, the second ICN innervated both the pectoralis major and minor, while the floor of the axilla and medial side of the arm were innervated by the ICBN emerging from the third ICS. The authors opined that an inadvertent lesion to these nerves during various axillary operating procedures may result in both sensory and motor loss [7]. On the other hand, in our study, ICBN was not observed to supply any muscle of the pectoral region. Whereas Loukas et al. presented a case of an 87-year-old female, in whom the right ICBN arose at its normal site within the right second ICS, traversed for around 3 cm, and received a unique medial pectoral branch emerging from the medial pectoral nerve. Awareness of these variances in conjunction with proper axillary dissection could prevent mastectomy patients from undesirable impairments, regardless of being sensory or motor [8]. However, unlike the previous report, the present study did not recognise any communication between the medial pectoral nerve and the ICBN. Consequently, inadvertent injury to the ICBN in such cases is less likely to be associated with motor deficits.

Furthermore, Borthakur et al. described an occurrence of abnormal bilateral ICBNs arising from the first ICNs in a 69-year-old male cadaver. The ICBN was connected with MCNA on the right side and with both MCNA and MCNF on the left side. Such an abnormal ICBN has numerous diagnostic ramifications in interpreting pain and paraesthesia, particularly in cases of intercostal neuralgia caused by entanglement, injury, or herpes zoster infection [9]. However, we observed the interconnecting ICBNs arising from the second and third ICS without any communication with MCNA. The occurrence of this interconnection between the ICBNs may pose an additional challenge for their safeguarding during various axillary surgeries.

Moreover, Supriya et al. discovered several ICBNs emerging from the first, second, and third ICS. These ICBNs were observed to innervate the medial and posterior regions of the arm, in addition to the MCNA and the posterior cutaneous nerve, and no connecting branch was noticed among them. The substantial cutaneous innervation to the arm by these ICBNs and their variant course offer valuable information to surgeons and oncologists during a variety of axillary surgical approaches [10]. Similarly, during dissection of a 50-year-old male cadaver, Xaviour observed three ICBNs originating from the first, second, and third ICS of the right side, which supplied the medial side of the arm. Moreover, the MCNA was not present in this unique cadaver, which was substituted by branches from the MCNF [11]. However, in the present case, we observed the normally originating ICBN from the right second ICS, the variant ICBN from the right third ICS, and an additional presence of the common trunk for the MCNA and MCNF (Table 1). Knowledge of the variant origin of the ICBN with the additional occurrence of the combined trunk for the MCNA and the MCNF can be vital for diverse operative procedures like axillary lymph node dissection, breast augmentation, brachioplasty, and orthopaedic surgeries of the upper arm and pectoral region.

**Table 1:** Comparison of origins, branches and communications of the ICBN with other studies ICBN: intercostobrachial nerve; MCNA: medial cutaneous nerve of arm; MCNF: medial cutaneous nerve of forearm; ICS: intercostal space

Researchers	Variant side	Origin	Branches	Communication with MCNA	Associated variation
Rustagi et al. [6]	Right	Second ICS	Not reported	Present	In addition to the ICBN, two nerves, ICBN1 and ICBN2, arose from the right second ICS
Singh et al. [7]	Bilateral	Right side: Second ICS; Left side: Third ICS	Right side: upper and lower branch; Left side: Not reported	Not reported	Right ICBN was carrying both the motor and sensory parts; on the left side, the second intercostal nerve innervated both the pectoralis major and minor
Loukas et al. [8]	Right	Second ICS	Not reported	Not reported	Right ICBN received a unique medial pectoral branch emerging from the medial pectoral nerve
Borthakur et al. [9]	Bilateral	First ICS	Right side: 2 rami; Left side: 3 rami	Present	Left ICBN was connected with both MCNA and MCNF
Supriya et al. [10]	Right	First, second and third ICS	T1 ICBN: 2 branches; T2 ICBN: 2 branches; T3 ICBN: undivided	Absent	Absent
Xaviour [11]	Right	ICBN 1, 2, and 3 from first, second and third ICS, respectively	Not reported	Absent	Median nerve had two lateral roots; absence of the MCNA was compensated by branches from the MCNF
Present study	Right	Second and third ICS	T2 ICBN: upper and lower branch; T3 ICBN: Single trunk	Absent	A common trunk for MCNA and MCNF was observed, which split at the middle of the arm

During embryonic development, the upper limb buds develop from the lateral body wall opposite the C4 to T2 somites, usually by the fourth week. With differentiation of the limb segments, the overlying surface ectoderm maintains a segmental innervation reflecting the embryological origin of each dermatome. Any deviation from this process may lead to variations in the cutaneous innervation of the upper limb [12].

## Conclusions

The ICBN is a recognisable anatomical structure having substantial variations in its origin, course, communications, and branching configurations. A variant origin of the ICBN from the third ICS communicating with the normally originating ICBN from the second ICS and an additional occurrence of the common trunk for MCNA and MCNF, as observed in the present case, may create considerable hazard during operative procedures in the axilla and upper arm. Surgeons should take all possible measures to safeguard the ICBN, its various subdivisions, and communicating fibres. Comprehensive knowledge of such variant cases of origin and communication of ICBN, combined with cautious surgical technique, can potentially minimise the risk of postoperative sensory disturbances during various axillary operating procedures encompassing mastectomy, breast and axillary reconstruction, axillary lymph node dissection, and axillary vascular surgeries.
